# Metronome Cueing of Walking Reduces Gait Variability after a Cerebellar Stroke

**DOI:** 10.3389/fneur.2016.00084

**Published:** 2016-06-01

**Authors:** Rachel L. Wright, Joseph W. Bevins, David Pratt, Catherine M. Sackley, Alan M. Wing

**Affiliations:** ^1^School of Sport, Exercise & Rehabilitation Sciences, University of Birmingham, Birmingham, UK; ^2^School of Psychology, University of Birmingham, Birmingham, UK; ^3^Institute of Sport and Exercise Science, University of Worcester, Worcester, UK; ^4^West Midlands Rehabilitation Centre, Birmingham Community Healthcare Trust, Birmingham, UK; ^5^Faculty of Life Sciences & Medicine, King’s College London, London, UK

**Keywords:** gait ataxia, auditory cueing, gait variability, gait kinematics, cerebellar stroke, rhythmic auditory stimulation

## Abstract

Cerebellar stroke typically results in increased variability during walking. Previous research has suggested that auditory cueing reduces excessive variability in conditions such as Parkinson’s disease and post-stroke hemiparesis. The aim of this case report was to investigate whether the use of a metronome cue during walking could reduce excessive variability in gait parameters after a cerebellar stroke. An elderly female with a history of cerebellar stroke and recurrent falling undertook three standard gait trials and three gait trials with an auditory metronome. A Vicon system was used to collect 3-D marker trajectory data. The coefficient of variation was calculated for temporal and spatial gait parameters. SDs of the joint angles were calculated and used to give a measure of joint kinematic variability. Step time, stance time, and double support time variability were reduced with metronome cueing. Variability in the sagittal hip, knee, and ankle angles were reduced to normal values when walking to the metronome. In summary, metronome cueing resulted in a decrease in variability for step, stance, and double support times and joint kinematics. Further research is needed to establish whether a metronome may be useful in gait rehabilitation after cerebellar stroke and whether this leads to a decreased risk of falling.

## Introduction

Cerebellar infarction accounts for ~3% of strokes, resulting in an incidence of ~20,000 cases per year in the USA (1). Gait instability and gait ataxia are reported in over half of those who experience an infarct in the cerebellum (2). Gait ataxia is associated with increased variability in both step time and step length (3), and although similar range of motion patterns were observed at the hip, knee, and ankle, the variability of these patterns were significantly higher for hip and knee motion compared to controls (4). These increases in variability are likely to be caused by the interaction between the stroke-induced deficits in balance control, limb control and coordination, and the resultant adjustments needed to respond to these deficits (5).

Any increase in the variability of gait parameters is of concern as a small increase in stride time variability is associated with a risk of future falls in community-dwelling older adults (6). Stance and swing time variability distinguishes between fallers and non-fallers (7), and greater stance time variability is associated with future mobility disability (8). Greater variability in step length and double support time is linearly associated with increased risk of multiple falls in older adults, with a non-linear association for step time variability (9). Both increased stride time and length variability are also associated with falls in cerebellar ataxia (10). The occurrence of falls can lead to an associated fear of falling, which can result in a reduction in activity and community involvement (11). Additionally, a high fear of falling also appears to place an individual at an increased risk of experiencing future falls (12). Therefore, reducing excessive gait variability resulting from a cerebellar stroke is an important concern for health-care professionals.

Temporal and spatial parameters, although providing useful information, only provide global measures of gait function. They do not provide information about potential changes at the hip, knee, and ankle joints. The variability of these joint kinematics are only infrequently reported; however, increased variability has been previously reported for individuals with multiple sclerosis (13) and Parkinson’s disease (14), as well as in gait ataxia (4). Therefore, the inclusion of joint angle variability in an assessment of gait variability will provide further information of gait function than by temporal and spatial parameters alone.

Research has shown that auditory rhythm and music can produce an effect on the motor system. Musical rhythms and auditory cues can facilitate muscle activation through the audio-motor pathways at the reticulospinal level (15), and studies have demonstrated the ability to synchronize lower limb movements to auditory cues (16, 17). Tapping studies have identified that a complex circuit involving different cortical areas, cerebellum and basal ganglia, are associated with timing and rhythmic processing (18). The posterior superior temporal gyrus and premotor cortex have been shown to be involved in the entrainment of movement to auditory cues (19). It has been suggested that although cerebellar damage may impair the conscious detection of rhythmic variation, it does not appear to effect motor entrainment to rhythmic stimuli (18). Therefore, the use of rhythmic stimuli for cueing movement may be a useful tool in the rehabilitation of movement deficits following a cerebellar stroke.

The use of an auditory cue for walking, such as a metronome, has been used for a variety of patient groups, such as those with Parkinson’s disease (20–22), post-stroke hemiparesis (23–26), and Huntingdon’s disease (27). For individuals with Parkinson’s disease, the use of a metronome reduced stride time and swing time variability, but only when the metronome was set to 10% higher than the preferred cadence, with the improvements still present 15 min later (21). For individuals with post-stroke hemiparesis, the use of a metronome set to each participant’s preferred cadence reduced paretic step time variability when stepping in place (28).

These studies indicate that the use of a metronome to cue gait can be a useful tool in reducing excessive gait variability; however, no study has investigated the use of a metronome with someone after a cerebellar stroke. Therefore, the aim of this case report was to investigate whether metronome cueing of gait produces an immediate effect on gait variability after a cerebellar stroke.

## Methods

The participant gave written and informed consent prior to the start of the experimental session. Favorable ethical opinion for the study was granted by the local Research Ethics Committee and was carried in accordance with the principles laid down by the Declaration of Helsinki.

### Case Description

A community-dwelling female participant (aged 81 years, height 1.60 m, mass 62.1 kg, Rivermead Gross Motor Function = 10, Fugl-Meyer lower limb score = 31 with slight dysmetria observed, International Cooperative Ataxia Rating Scale for gait and posture disturbances = 11) volunteered for this study. Twelve months prior to testing, she was diagnosed with a left cerebellar infarct involving the left posterior inferior cerebral artery (PICA) by CT scan. She had also been diagnosed with atrial fibrillation, and at the time of testing was medicated with metoprolol, digoxin, and cardioplen.

She described her walking style as “feeling like I am drunk, but I am not” and “I feel like I lurch when I walk.” She stated that she found walking tiring. She had been given an appropriate stick to walk with by a physiotherapist, but preferred not to use it. She had a history of recurrent falling since her stroke and described falls as initially occurring forwards, but they resulted with impact on her side. The recurrent falling had resulted in a high fear of falling [Falls Efficacy Scale – International = 49 (29, 30)], with activities, involving walking, evoking the highest concern responses. This high fear of falling resulted in the participant limiting her activities outside the home to when she was accompanied by her spouse.

### Gait Assessment

Whole-body motion data were collected at 60 Hz using a 15-camera Vicon system (Vicon Peak, Oxford Metrics Ltd., UK) set up in a large (17 m × 12 m × 4.5 m) gait laboratory. The full-body Vicon Plug-in Gait (PiG) marker set was used. Custom written software running on a laptop provided clearly audible metronome pulses. The metronome generated a voltage pulse that was passed to the Vicon system to allow integration with the motion capture data.

The participant familiarized herself with walking in the laboratory and then performed three gait trials at her comfortable pace. Her cadence was averaged from these trials to generate the inter-beat interval for the metronome. The participant then had a 5-min familiarization period to the metronome. After the familiarization period, a further three gait trials were performed with metronome cueing. The metronome was commenced prior to the initiation of walking, and the participant started to walk when she was ready. The first three steps and last three steps of each walking trial were discarded to allow for acceleration and deceleration.

### Data Analysis

According to typical practice for gait analysis studies, data were combined from individual walking trials for each condition. This allowed 24 steps of data to be collected for analysis for each condition, meeting the recommendations for gait variability studies (31). The coefficient of variation (CoV) was calculated as a measure of gait variability for the temporal and spatial gait parameters for the left and right sides.

Marker position data were filtered using the Woltring cross-validity quintic spline routine (32) to minimize marker trajectory noise. Sagittal hip, knee, and ankle angles were calculated from the kinematic marker data using the PiG model (Vicon Peak, Oxford Metrics Ltd., UK) and reported in degrees. Joint angles were segmented into discrete gait cycles and normalized to 0–100% of the gait cycle. Means and SDs of the joint angles were calculated at each percent of the gait cycle. The mean of these SDs were then calculated to give a measure of variability of joint motion (13, 33).

## Results

The participant’s normal walking speed was 0.68 m s^−1^, reducing slightly to 0.65 m s^−1^ when walking to the metronome. Heel strike occurred 0.04 ± 0.02 s ahead of the leading edge of the metronome impulse. The CoV was reduced from 9.9 to 3.8% and 10.9 to 3.3% for left and right step times, respectively (see Table 1), from 7.2 to 4.5% (left) and from 8.0 to 6.8% (right) for stance times and from 18.7 to 14.8% (left) and from 18.8 to 14.2% (right) for double support times when walking in the metronome-cued condition compared to baseline. The CoV for left and right step lengths was also slightly reduced when walking to the metronome.

**Table 1:** **Temporospatial gait parameters with and without the metronome**.

		Without metronome			With metronome		
		Mean	SD	CoV	Mean	SD	CoV
Walking speed (m s^−1^)		0.68	–	–	0.65	–	–
Step length (m)	Left	0.49	0.04	8.17	0.50	0.03	6.95
	Right	0.47	0.04	9.40	0.49	0.04	7.72
Step time (s)	Left	0.65	0.06	9.93	0.67	0.03	3.84
	Right	0.66	0.07	10.92	0.67	0.02	3.26
Stance time (s)	Left	0.90	0.07	7.23	0.89	0.04	4.49
	Right	0.95	0.08	7.94	0.91	0.06	6.78
Swing time (s)	Left	0.43	0.04	8.36	0.43	0.04	8.28
	Right	0.40	0.02	4.45	0.41	0.04	8.80
Double support time (s)	Left	0.24	0.05	18.71	0.24	0.04	14.78
	Right	0.24	0.04	18.81	0.23	0.03	14.18

**Figure 1:**
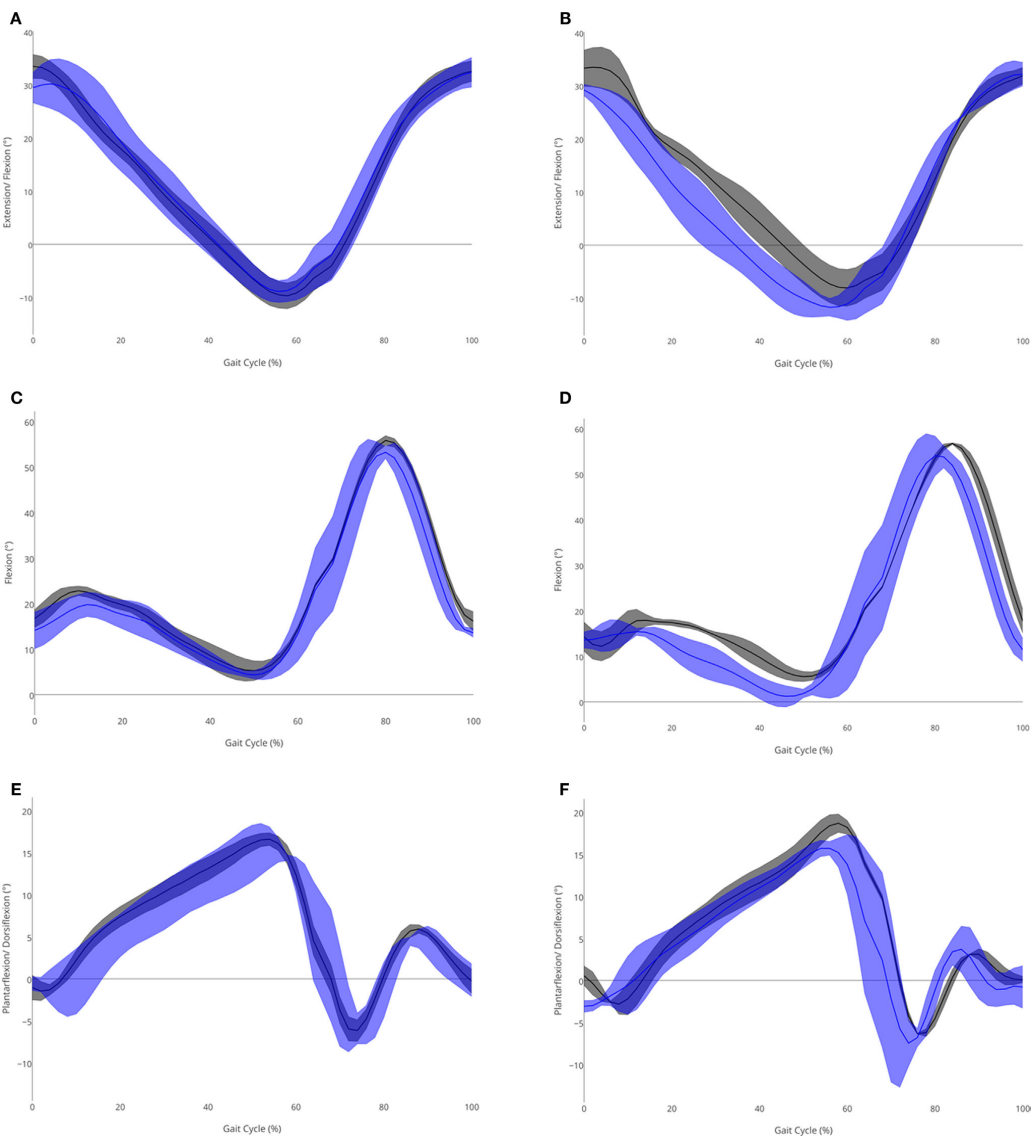
**Sagittal joint angles (°) in the metronome (gray) and no metronome (blue) conditions for (A) left hip, (B) right hip, (C) left knee, (D) right knee, (E) left ankle, and (F) right ankle**. Means (solid lines) and SDs (shaded area) shown.

There were reductions in the variability of the sagittal joint motion patterns at the hip, knee, and ankle (see Table 2), with the largest decreases in variability occurring in knee motion (baseline left SD = 3.8, right SD = 4.3; metronome left SD = 1.5, right SD = 1.5). This was particularly apparent during the stance phase of the gait cycle for hip motion (see Figures 1A,B), and during the swing phase for knee (Figures 1C,D), and ankle motion (Figures 1E,F).

**Table 2:** **Sagittal joint angles (°) with and without the metronome**.

		Without metronome		With metronome	
		Left	Right	Left	Right
Hip	Peak flexion	32.6	32.2	33.5	33.5
	Peak extension	−8.9	−11.7	−9.1	−8.0
	Variability (mean SD)	4.0	3.7	2.3	2.8
Knee	Peak flexion in stance	19.8	15.4	22.8	17.9
	Peak flexion in swing	53.2	54.0	55.8	56.7
	Variability (mean SD)	3.8	4.3	1.5	1.5
Ankle	Peak dorsiflexion	15.6	15.7	16.6	18.7
	Peak plantarflexion	−5.9	−7.5	−6.2	−6.3
	Variability (mean SD)	2.7	2.6	1.2	1.2

## Discussion

The aim of this study was to investigate whether using an auditory metronome to cue gait could reduce excessive gait variability in a participant who had experienced a cerebellar stroke. Gait ataxia is reported in over half of those with a cerebellar infarct in the PICA territory (2), and the participant had a history of repeated falls since her stroke. Increased stride time and step time variability are associated with a risk of future falls in older adults (6, 9) and are associated with falls history in cerebellar ataxia (10). This participant demonstrated higher step time variability at baseline (left 9.9%; right 10.9%) than would be considered normal (5.5 ± 2.6%) for a woman of her age (34). Cueing by the metronome resulted in a large decrease in step time variability (left 3.8%; right 3.3% with cueing) and reduced these values to within a normal range without any significant changes to step times.

The participant displayed higher step length variability at baseline than the normal values previously reported for her age group (34). Increased variability in step length and double support time is linearly associated with an increased risk of multiple falls in older adults (9). Therefore, the decreases in variability of these parameters when walking to the metronome in this participant may reduce her risk of multiple falls if the decreased variability is maintained in everyday walking. Stance time variability during uncued walking was at a level associated with prevalent mobility disability (35). Stance time variability was also reduced when walking to the metronome, with a decrease in the SD of greater than the 0.01 s previously reported as a clinically meaningful reduction in variability during walking for older adults (36).

The participant in this study displayed normal elderly joint kinematics during walking (37). However, she has higher joint angle variability across the hip, knee, and ankle than has been previously reported for healthy controls and individuals with multiple sclerosis (13), and at the hip and knee than has previously been reported for older adults (33). When walking was cued by the metronome, the variability was decreased in all joint angle measures. With the exception of the right hip, the joint variability measures are within the range of previously reported healthy controls (13) when cued by the metronome.

The participant’s reports of falls impacting on the side are a cause for concern, as falls impacting on the side (and therefore in the region of the greater trochanter) are more likely to result in a hip fracture than a forward or backward fall (38, 39). Therefore, reducing fall incidence is of primary concern to reduce the risk of serious injury. Participant fatigue prevented any investigation of an immediate carry-over effect of the metronome on reducing gait variability, and whether a reduction in gait variability also resulted in reduced fall occurrence in this participant is beyond the scope of this study. However, any potential reduction in fall incidence may also reduce the participant’s fear of falling allowing her to be less dependent on company to leave the house. A low fear of falling is thought to have a protective effect against fall occurrence (12). Therefore, future research is required on the use of rhythmic cues to reduce excessive gait variability in groups prone to falling.

The motor system is very sensitive to stimulation by the auditory system (15), and studies have demonstrated the ability to time footfalls to auditory cues (16, 17). The coupling of footfalls to acoustic pacing has been termed auditory-motor anchoring, and this influences coordinative stability and movement variability at this phase of the gait cycle (24, 40). Auditory cueing of gait has been investigated in a variety of different pathologies and has been proposed as one of the most promising approaches to improve gait coordination in post-stroke hemiparesis (41). Therefore, auditory cueing is worthy of investigation in other pathologies affecting gait, such as ataxia after cerebellar stroke.

This case report suggests that the use of a rhythmic auditory cue, such as a metronome, produces an immediate effect on excessive gait variability in a participant with cerebellar stroke. Future research is warranted to ascertain whether this is replicated in other participants after a cerebellar stroke and to establish whether the benefits can be maintained using a rhythmic cue as a rehabilitation technique such as a rhythmic auditory stimulation stepwise limit cycle entrainment protocol (26).

## Author Contributions

Conceived and designed the experiments: RW, DP, CS, and AW. Data collection and analysis: RW and JB. Wrote the manuscript: RW. Critical revision of manuscript: JB, DP, CS, and AW. All the authors read and approved the final manuscript.

## Conflict of Interest Statement

The authors declare that the research was conducted in the absence of any commercial or financial relationships that could be construed as a potential conflict of interest.
